# StrongerSORT: Improving DeepSORT for Stronger Human Tracking

**DOI:** 10.3390/s26185878

**Published:** 2026-09-17

**Authors:** Yinuo Wang, Xinlu Zhong, Jiayi Guan, Da Lv, Jintao Sheng, Yunhua Tan, Yali Zheng

**Affiliations:** 1 School of Automation Engineering, University of Electronic Science and Technology of China, Chengdu 611731, China; 202322280429@std.uestc.edu.cn (Y.W.); 202421060130@std.uestc.edu.cn (J.G.); 202521060323@std.uestc.edu.cn (D.L.); 202422060132@std.uestc.edu.cn (J.S.); 2 Shenzhen Institute for Advanced Study, University of Electronic Science and Technology of China, Chengdu 610042, China; 3 Dongfang Boiler Group Co., Ltd., Dongfang Electric Corporation, Chengdu 611730, China; zhongxl6642@dongfang.com (X.Z.); tanyh@dbc.com.cn (Y.T.)

**Keywords:** human detection and tracking, video monitoring system, multiple-object tracking

## Abstract

Human tracking plays a crucial role in video surveillance systems. However, tracking humans in surveillance videos remains challenging because targets are often captured at long distances, occupy only a small number of pixels, and exhibit substantial scale variations. These challenges require not only accurate detection of small, low-texture targets but also fast and robust data association for multi-object tracking. We propose an enhanced human-tracking method that integrates improved object detection with complementary appearance and motion cues. Specifically, we improve YOLOv12 by incorporating large-kernel deformable attention, dynamic convolution, and phantom convolution. These components enhance the detector’s ability to perceive small-target features under complex backgrounds and occlusion while reducing its computational cost. The OSNet appearance embeddings incorporated into the EMA update framework construct a robust trajectory-level temporal appearance representation, which improves the discrimination between different individuals in the tracking stage. Extensive experiments demonstrate that the proposed method achieves more accurate identity association and more stable target trajectories than state-of-the-art tracking methods, including StrongSORT and ByteTrack.

## 1. Introduction

Human detection and tracking have long been important research topics and have witnessed rapid advances in vision-based sensing and perception [1,2]. Deep learning-based detection and tracking methods have been extensively investigated and widely applied [3,4,5,6,7]. Nevertheless, several challenges remain. First, conventional detectors often exhibit limited recall and localization accuracy for distant and small-scale targets, particularly when substantial scale variations occur. Such detection deficiencies provide unstable observations for subsequent tracking, thereby increasing errors in personnel counting and event recognition. Second, existing models often require substantial computational resources to operate effectively in complex scenarios. Simplifying these models to meet real-time processing requirements frequently compromises detection performance, resulting in a persistent trade-off between accuracy and computational efficiency. Third, in densely populated scenarios involving frequent interactions and severe occlusions, even minor localization errors or brief missed detections can undermine data association, leading to increased trajectory fragmentation and identity switches. Therefore, achieving accurate detection of distant, small-scale, and low-texture targets while maintaining fast and robust multi-object tracking remains a significant challenge.

The proposed method follows a two-stage multi-object tracking framework. In the first stage, an enhanced YOLOv12 detector is employed to detect human targets in each frame. In the second stage, motion and appearance information is jointly exploited for data association, thereby generating continuous trajectories for individual targets. The main contributions of this work can be summarized as follows. First, an enhanced detector based on YOLOv12 [8] is developed to alleviate missed detections and localization instability caused by small target sizes, background interference, and occlusion. Specifically, deformable large-kernel attention is incorporated into the feature extraction module to enhance the network’s ability to capture discriminative features of small targets in complex backgrounds and under occlusion. Second, dynamic convolution and Ghost convolution are introduced to reduce the computational complexity of the detector while preserving its detection accuracy, thereby improving its suitability for deployment on edge devices. Third, OSNet is employed as the appearance feature extractor to improve the discriminability of appearance representations among visually similar targets. This design enhances cross-frame association accuracy and reduces the occurrence of identity switches. Overall, the enhanced YOLOv12 detector provides more reliable observations for the subsequent tracking stage, the OSNet appearance embeddings incorporated into the EMA update framework construct a robust trajectory-level temporal appearance representation, resulting in fewer missed detections, more accurate data association, and improved overall tracking performance.

## 2. Related Work

### 2.1. Object Detection

Research on object detection has long attracted attention from both academia and industry. Its development has generally progressed from traditional manual feature methods to deep learning methods, gradually forming two main branches: two-stage and end-to-end methods.

Traditional object detection methods rely heavily on manually designed features, making them sensitive to environmental factors such as lighting changes, occlusions, and background clutter. They typically follow a paradigm of “region proposal generation + feature extraction + classifier decision”. A typical pipeline consists of image preprocessing, followed by acquiring candidate regions through sliding windows, image pyramids, or region proposal generation. These regions are represented using hand-crafted features such as HOG, Haar, or LBP or structural features like edges. Classifiers such as SVM or AdaBoost are employed to perform object discrimination and localization refinement [9,10,11,12]. However, in general, traditional methods have problems such as limited feature representation ability, insufficient generalization ability, reliance on human experience, and high computational cost, making them difficult to adapt to complex scenarios and large-scale data requirements.

The introduction of deep networks such as AlexNet [13], VGG [14], and ResNet [15] enabled the two-stage detector to achieve a substantial improvement in detection accuracy. Two-stage detectors typically offer greater accuracy, especially in scenarios with small targets, occlusion, and complex backgrounds. However, their inference process is more complex and their latency is relatively high, which often limits their performance in strictly real-time scenarios. Unlike the two-stage approach of first proposing candidates and then classifying them, one-stage detectors treat detection as a dense prediction problem. By directly regressing bounding boxes and class probabilities on the feature maps, they offer the advantages of simple structure and fast speed, and have therefore gradually become the mainstream approach for real-time object detection. YOLO, proposed by Redmon et al. [16], was the first to systematically incorporate a one-stage end-to-end real-time detection framework. It outputs the class labels and box position in one forward propagation through gridded regression, achieving significant real-time performance [16]. However, its accuracy is insufficient in dense target and small target scenarios due to the grid mechanism limitation. Thanks to the collective work of researchers, YOLO has been iterated repeatedly. The YOLOv12 proposed by Tian et al. [8] shows stronger overall competitiveness compared to mainstream real-time detectors of the same period on benchmarks such as COCO, while maintaining low-latency inference characteristics [8]. Its attention mechanism enhances the modeling of small targets and fine-grained local features, which is beneficial to improving detection reliability. Furthermore, its low latency and engineering-friendly architecture facilitate deployment on edge GPUs and allow for the formation of a high-frame-rate ’detection-association’ pipeline with subsequent multiple object tracking modules, greatly meeting the system’s comprehensive requirements for real-time performance, accuracy, and portable deployment.

### 2.2. Multiple-Object Tracking

Research on multiple-object tracking mainly relied on generative modeling, using motion models to recursively deduce target states and perform inter-frame correlation. Typical works include the Kalman filter proposed by Kalman [17], the particle filter proposed by Arulampalam et al. [18], the optical flow method proposed by Horn and Schunck [19], and the MeanShift method proposed by Comaniciu et al. [20]. These methods are simple to implement and have low computational overhead. However, due to their limited ability to distinguish appearance and their sensitivity to complex backgrounds, dense occlusions, and nonlinear motion, they are prone to tracking loss and frequent ID switches in complex scenarios, making it difficult to meet the requirements for high accuracy and high reliability.

With the improvement in deep learning–based detectors, MOT has gradually developed a technical paradigm centered on detection and association, evolving along two main directions: tracking-by-detection (DBT) and end-to-end joint modeling (end-to-end, Transformer-based MOT). In recent years, within the DBT framework, MOT has continuously enhanced occlusion recovery and association robustness through methods such as ByteTrack [21], BoT-SORT [22], OC-SORT [23], and Hybrid-SORT [24]. Wojke et al. proposed DeepSORT [25], which introduces ReID appearance features [26] and a cascade matching strategy into the SORT framework, significantly improving identity preservation in occlusion scenarios and becoming a highly influential classical baseline in engineering applications. End-to-end MOT continues to advance NMS-free and unified modeling, but engineering implementation still faces stability and cost constraints. For the task in this paper, the system constraints place greater emphasis on real-time online performance, ease of deployment and portability, and long-term ID stability. The tracking module needs to be decoupled from the detector, enabling rapid replacement of detection models and parameter tuning across different edge computing platforms. At the same time, it needs to minimize ID switches under occlusion and personnel interactions, ensuring reliable counting and trajectory backtracking.

## 3. Method

### 3.1. Overall Framework

**Figure 1:**
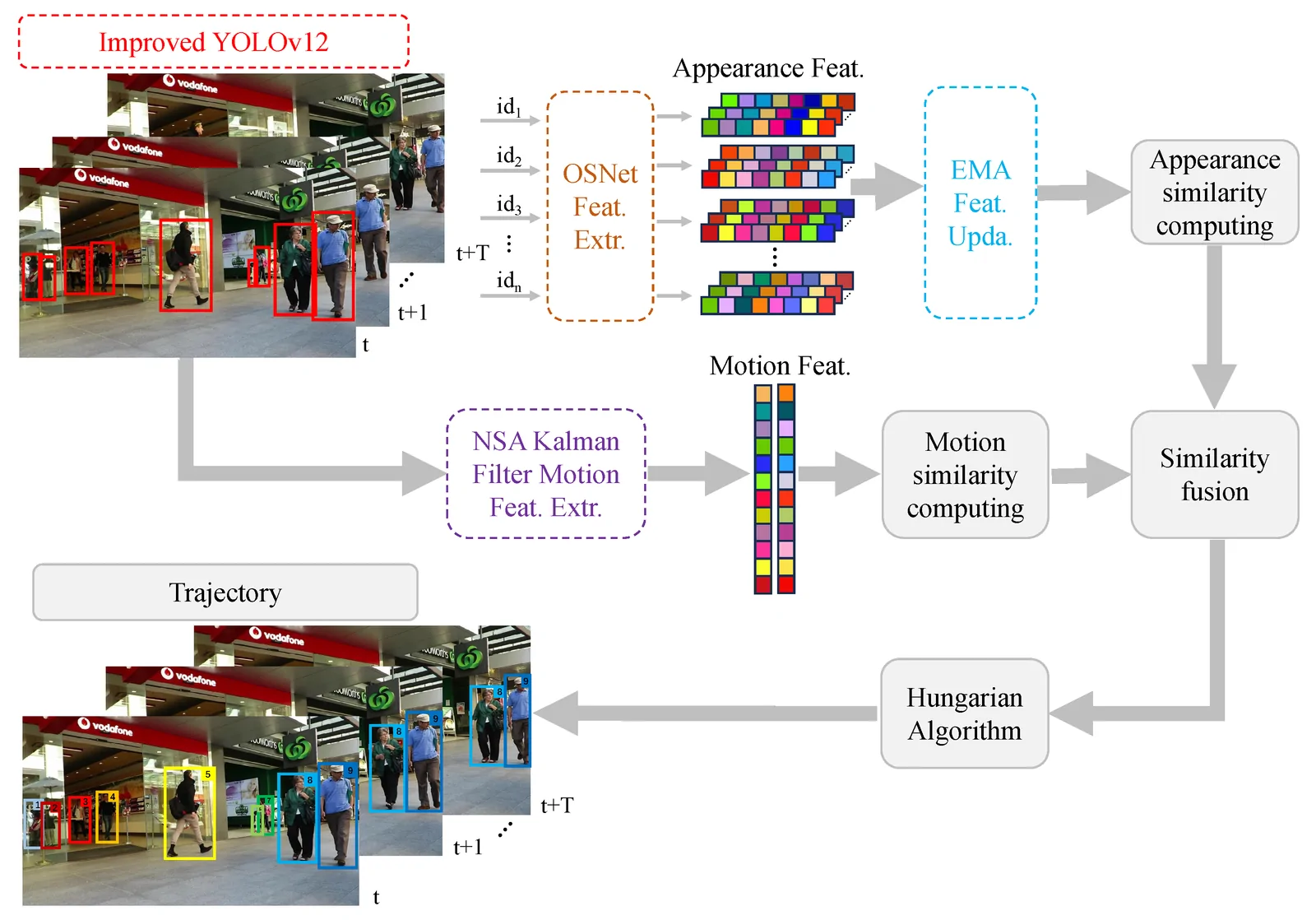
The Framework for Human trajectory tracking.

The proposed human-tracking framework is illustrated in Figure 1. The overall procedure consists of the following four steps. (1) The input video is processed frame by frame to generate a sequence of images. For each frame, the enhanced YOLOv12 detector is employed to detect human targets and produce their bounding boxes and confidence scores. (2) Each detected target region is cropped according to its bounding box and fed into OSNet to extract an appearance feature vector. Meanwhile, based on the historical states of each track, the NSA Kalman filter predicts the target’s current position and motion state. (3) The appearance and motion distances between the detections in the current frame and the existing tracks are then computed. Specifically, the appearance distance is measured using cosine distance, whereas the motion distance is measured using Mahalanobis distance. These two distance measures are subsequently fused to construct an association cost matrix between the current detections and the existing tracks. The Hungarian algorithm is then applied to determine the optimal assignment, thereby associating the detected targets in the current frame with the corresponding historical tracks. (4) Finally, the track states and target identities are updated according to the association results, enabling continuous trajectory estimation and identity preservation for human targets throughout the video and producing stable multi-object tracking results.

### 3.2. Human Detection Based on Improved YOLOv12

**Figure 2:**
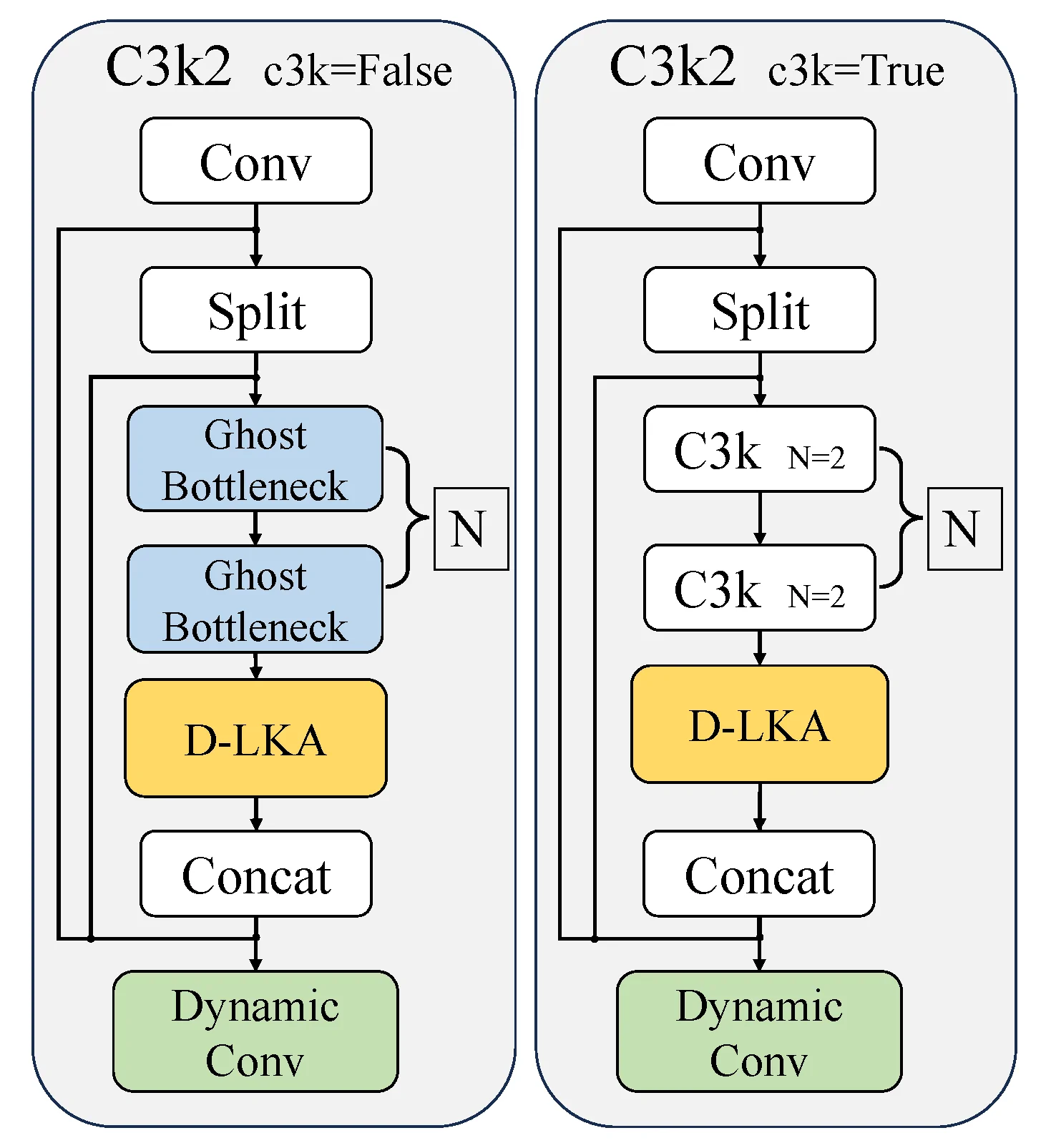
Improved YOLOv12 modules for human detection.

In the backbone of YOLOv12, the C3k2 module still has certain limitations when handling highly varying texture details. Its fixed convolutional kernels may cause attention dispersion and partial loss of fine-grained information, leading to missed detections or localization errors in dense small-object scenarios. To overcome these limitations, the proposed method improves the detector from three aspects: network structure, model lightweighting, and the loss objective function. First, a joint mechanism of large kernel convolution and deformable convolution is introduced into the C3k2 module to construct the Deformable-LKA attention module. The proposed module is then deployed in the Backbone and Neck to expand the effective receptive field and enhance the spatial adaptive modeling capability, thereby improving the accuracy and robustness of small target detection. Second, lightweight strategies such as Ghost Conv and Dynamic Conv are introduced to enhance feature representation and improve real-time inference efficiency without increasing the number of parameters and computational overhead. The improved C3k2 module network structure is shown in Figure 2. Finally, an weighted regression loss [27] is used to optimize the bounding box regression process, following Equation (1), to improve localization accuracy and enhancing training convergence stability. The WIoU mechanism is employed to modulate the CIoU regression loss. CIoU serves as the primary geometric regression objective, while WIoU’s distance-based attention and dynamic non-monotonic focusing mechanism act as a sample-level weighting gate to adaptively redistribute regression gradients. The distance-based attention component intensifies regression for samples with significant center offsets, accelerating convergence during the early stages of training. Meanwhile, the dynamic non-monotonic focusing mechanism highlights the learning contribution of medium-quality samples and suppresses extreme gradients from low-quality outliers and high-quality easy samples, thereby enhancing training stability and generalization capability.

(1) $$L_{\mathrm{reg}}=r\left(\beta \right)R_{\mathrm{WIoU}}\cdot L_{\mathrm{CIoU}},$$

where $\beta =\frac{L_{\mathrm{IoU}}}{\bar{L_{\mathrm{IoU}}}}$, r(β) is a Dynamic Non-Monotonic Focal function, $L_{\mathrm{CIoU}}=1-\mathrm{CIoU}$, $R_{\mathrm{WIoU}}=\exp\left(\frac{{\left(x-x_{gt}\right)}^{2}+{\left(y-y_{gt}\right)}^{2}}{W_{g}^{2}+H_{g}^{2}}\right)$.

### 3.3. OSNet-Based Discriminative Feature Extraction Module

In order to improve the discriminative capability and robustness of appearance representation in multiple object tracking, the Omni-Scale Network (OSNet) [27] is introduced to replace the original BoT-based appearance feature extractor in StrongSORT. We adopt OSNet as the backbone network for human re-identification (ReID) in the appearance feature extraction module. The core design of OSNet is full-scale feature learning. It constructs multi-scale branches within the same residual block to extract features of different receptive fields, and dynamically fuses these multi-scale features using a unified aggregation gate (AG), thereby achieving more stable identity discrimination under fine-grained differences. For input features x, the residual block can be written as

(2) $$y=x+\tilde{x},\;\tilde{x}=\sum_{t=1}^{T}F_{t}\left(x\right)$$

where $F_{t}$ represents the mapping function of the *t*-th scale branch, and *T* is the number of scale branches, usually T=4, covering multi-scale receptive fields from local to global. After gating fusion

(3) $$\tilde{x}=\sum_{t=1}^{T}G\left(x_{t}\right)⊙x_{t}$$

where G(·) generates a weight vector consistent with the number of channels, and ⊙ represents the channel-by-channel product.

The advantages of OSNet are mainly reflected in two aspects. First, full-scale feature learning captures both local details and global structure, while the aggregation gate (AG) adaptively fuses multi-scale features to improve identity consistency under occlusion, viewpoint variations, and the presence of similar-looking pedestrians. Second, the lightweight design reduces model size and computational cost, making it suitable for real-time monitoring or edge deployment, and reducing the risk of overfitting on small to medium size ReID datasets. Downstream data association stage, OSNet typically outputs a 512-D feature vector from the final fully connected layer for cross-frame identity association.

### 3.4. Motion State Estimation Module Based on Noise Adaptive Kalman Filter

The Kalman filter (KF) [17] is a classic recursive optimal state estimation method that provides minimum mean square error estimates of dynamic system states under noisy observations. In multiple-object tracking, KF is used to predict and update trajectories, providing smooth state estimates. However, traditional KF typically assumes that the measurement noise is constant and ignores the differences in confidence between different detection boxes. Consequently, low-confidence detections may lead to inaccurate state updates and trajectory instability in complex real-world scenarios, especially when the target is occluded, blurred, or a small target at a distance.

To address the above issues, we employ the NSA Kalman filter [28], introducing a measurement noise adaptive adjustment mechanism driven by detection confidence within the standard KF framework. Specifically, NSA-KF constructs an adaptive measurement noise covariance based on the detection confidence level $c_{k}\in \left[0,1\right]$ in the *k*-th frame. The formulation is as follows,

(4) $${\tilde{R}}_{k}=\left(1-c_{k}\right)R_{k}$$

where $R_{k}$ is the preset noise covariance. This mechanism enables the filter to rely more on the prediction model when the observation confidence is low and to trust the observations more when the confidence is high, thereby achieving adaptive adjustment of the update intensity. When $c_{k}$ is low, ${\tilde{R}}_{k}$ increases and the Kalman gain $K_{k}$ decreases accordingly. Consequently, the filter relies more on the motion model prediction during updates, which suppresses “over-correction” caused by false detections, bounding box jitter, or localization errors. Conversely, when $c_{k}$ is high, ${\tilde{R}}_{k}$ decreases and $K_{k}$ increases. The filter places greater trust in the observational data, allowing the trajectory to align more rapidly with the true target position. To enhance the matching between the predicted bounding box and the target, we parameterize the bounding-box width w and height h instead of the aspect ratio r and height h [25]. We adjust the state vector,

(5) $$x_{k}={\left(x_{c},y_{c},w,h,{\dot{x}}_{c},{\dot{y}}_{c},\dot{w},\dot{h}\right)}^{⊤}$$

The corresponding measurement vector is

(6) $$z_{k}={\left(x_{c},y_{c},w,h\right)}^{⊤}$$

The NSA-KF update phases are shown in Table 1.

Through the aforementioned adaptive mechanism, NSA-KF can dynamically adjust the measurement update based on the confidence level of the detection box. This mechanism suppresses trajectory jitter under low-quality observations while enabling the estimated target state to rapidly converge to the true target position under high-quality observations, thereby significantly enhancing the robustness and smoothness of multi-object tracking. Especially in small target tracking tasks such as helmets, this method effectively suppresses trajectory drift and ID switching problems caused by false detections, occlusion, and  illumination variations.

### 3.5. EMA-Based Appearance Feature Temporal Smoothing Update Mechanism

In multiple object tracking, the stability of appearance features is crucial for cross-frame association. DeepSORT uses a feature bank to store historical appearances and matches them using the least cosine distance, but it is susceptible to detection noise and incurs additional computational overhead. Therefore, we continue to employ the exponential moving average (EMA) mechanism in StrongerSORT to update the trajectory appearance online. Specifically, we construct a track-level temporal appearance representation by integrating the OSNet-extracted appearance embedding $f_{i}^{t}$ of the detection successfully associated with track *i* in frame *t* into its appearance state vector $e_{i}^{t}$ using an EMA-based updating strategy, as follows:

(7) $$e_{i}^{t}=\alpha \;e_{i}^{t-1}+\left(1-\alpha \right)\;f_{i}^{t}$$

where α is the momentum coefficient, typically set as 0.9. The EMA mechanism can smooth out noise detection while preserving historical appearance information, achieving stable feature representation. Based on the appearance state vector updated by EMA, the cost of matching the trajectory with the detected appearance can be directly calculated as follows,

(8) $$d_{\mathrm{app}}\left(i,j\right)=1-f_{j}^{⊤}e_{i}$$

Compared with the feature memory bank approach, EMA only needs to maintain a single appearance vector for each trajectory and reduce the matching process to a constant number of computations. This significantly lowers the time cost during the association stage while enhancing robustness to appearance jitter and detection noise.

### 3.6. Linear Global Cost Matrix-Based Data Association Optimization Strategy

In multiple-object tracking, the data association strategy directly impacts trajectory matching stability and identity consistency. To overcome the local optima issue potentially incurred by the cascaded matching mechanism of DeepSORT in dense object or severe occlusion scenarios, this paper adopts linear global matching. Specifically, all currently active trajectories and detection boxes are integrated simultaneously to formulate a bipartite graph matching problem, which is then solved via linear assignment to achieve the globally optimal association.

In the implementation, a Kalman prediction ${\hat{x}}_{k|k-1}$ along with its covariance $P_{k|k-1}$ is first generated for each trajectory. Subsequently, a gating mechanism is employed to filter out improbable association pairs, such as the gating based on Mahalanobis distance:

(9) $$d_{\mathrm{mah}}^{2}\left(i,j\right)={\left(z_{j}-H{\hat{x}}_{k|k-1}^{\left(i\right)}\right)}^{⊤}S_{k}^{-1}\left(z_{j}-H{\hat{x}}_{k|k-1}^{\left(i\right)}\right),\;S_{k}=HP_{k|k-1}^{\left(i\right)}H^{⊤}+R_{k}$$

Then, a joint cost matrix C is constructed by integrating both appearance and motion information:

(10) $$C=\lambda A_{a}+\left(1-\lambda \right)A_{m},$$

where $A_{a}$ denotes the appearance cost calculated via cosine distance, $A_{m}$ represents the motion cost computed via Mahalanobis distance, and λ∈[0,1] is a weighting factor utilized to balance their respective contributions. Given *N* trajectories and *M* detection boxes, the linear assignment problem can be formulated as follows:

(11) $$\min_{x_{ij}}\sum_{i=1}^{N}\sum_{j=1}^{M}C_{ij}\cdot x_{ij},\;s.t.\;x_{ij}\in \left\{0,1\right\},\;\sum_{j=1}^{M}x_{ij}\leq 1,\;\sum_{i=1}^{N}x_{ij}\leq 1$$

### 3.7. Implementation Details

The experiments were conducted on a Windows 11 operating system. The programming language used was Python 3.9.13, with PyTorch 2.6.0 as the deep learning framework. GPU acceleration was enabled using CUDA 12.4, with NVIDIA driver version 553.35. The hardware configuration consisted of an NVIDIA RTX A6000 GPU and an Intel(R) Xeon(R) Gold 6326 CPU @ 2.90 GHz. The training settings are shown in Table 2. Table 3 lists the network architecture configuration of the backbone that was used in this paper. Compared to the original YOLOv12n, we replace C3k2 in layers 3 and 5, and A2C2f in layers 7 and 9 with our proposed modules C3k2_DCNv3_DLKA in the backbone network. The input size is also 640 × 640, and the detection head structure remains the same.

## 4. Experiments

### 4.1. Evaluation Metrics

(1) MOTA—Used to comprehensively evaluate the overall accuracy of multi-object tracking, it mainly considers three types of errors: false negatives (FN), false positives (FP), and identity switches (IDs). This metric is sensitive to both detection and association errors, and MOTA can become negative when the total number of tracking errors exceeds the number of ground-truth targets.

(12) $$\mathrm{MOTA}=1-\frac{\mathrm{FN}+\mathrm{FP}+\mathrm{IDs}}{\mathrm{GT}}.$$

(2) IDF1 (Identification F1 Score)—Used to measure the accuracy of identity consistency, it is an F1 score calculated based on the precision and recall of correct identity matches. This metric focuses on whether cross-frame identity associations are correct, i.e., the degree of identity matching between predicted trajectories and ground-truth trajectories in the temporal dimension.

(13) $$\mathrm{IDF}1=\frac{2\cdot \mathrm{IDTP}}{2\cdot \mathrm{IDTP}+\mathrm{IDFP}+\mathrm{IDFN}}.$$

(3) HOTA (Higher Order Tracking Accuracy)—A comprehensive evaluation metric designed to measure both detection precision and association quality. Unlike MOTA, which only focuses on detection errors, HOTA unifies detection correctness and identity association correctness into its evaluation framework, enabling a more balanced reflection of the overall performance of the algorithm at both the “detection” and “association” levels.

(14) $$\begin{array}{ccc} & \mathrm{HOTA} & =\sqrt{\mathrm{DetA}\cdot \mathrm{AssA}},\\ where & \mathrm{DetA} & =\frac{\mathrm{TP}}{\mathrm{TP}+\mathrm{FN}+\mathrm{FP}},\\ & \mathrm{AssA} & =\frac{\mathrm{TPA}}{\mathrm{TPA}+\mathrm{FPA}+\mathrm{FNA}}. \end{array}$$

(4) ID Switches (IDs)—Used to measure the degree of disruption to target identity consistency during tracking. When the same ground-truth target is assigned different tracking IDs in adjacent frames, it is counted as one identity switch; the cumulative number of such switches in the sequence is IDs.

(5) Frag (Fragmentation)—Represents the number of trajectory fragmentations, used to measure how many times the same ground-truth target is split into multiple independent trajectory segments during tracking. A smaller Frag value indicates better trajectory continuity, meaning the target can be correctly recovered after occlusion or temporary loss, leading to stronger tracking stability.

(15) $$\mathrm{Frag}=\sum_{i=1}^{N}\left(k_{i}-1\right).$$

(6) MT (Mostly Tracked)—Represents the proportion of target trajectories that are successfully tracked for more than 80% of their true lifecycle. Meanwhile, ML (Mostly Lost) represents the proportion of target trajectories that are tracked for less than 20% of their true lifecycle.

(16) $$\mathrm{MT}=\frac{N_{\mathrm{MT}}}{N_{\mathrm{GT}}},\;\mathrm{ML}=\frac{N_{\mathrm{ML}}}{N_{\mathrm{GT}}}.$$

where $N_{\mathrm{GT}}$ is the total number of ground-truth trajectories, $N_{\mathrm{MT}}$ is the number of trajectories satisfying $r_{i}\geq 0.8$, and $N_{\mathrm{ML}}$ is the number of trajectories satisfying $r_{i}<0.2$.

### 4.2. Qualitative Results

In this paper, the MOTChallenge dataset series primarily serves as a unified benchmark platform for multiple-object-tracking evaluation. The training set is utilized for algorithm tuning and pipeline validation, while the test set is used for final performance reporting and fair comparisons with existing methods.

**Figure 3:**
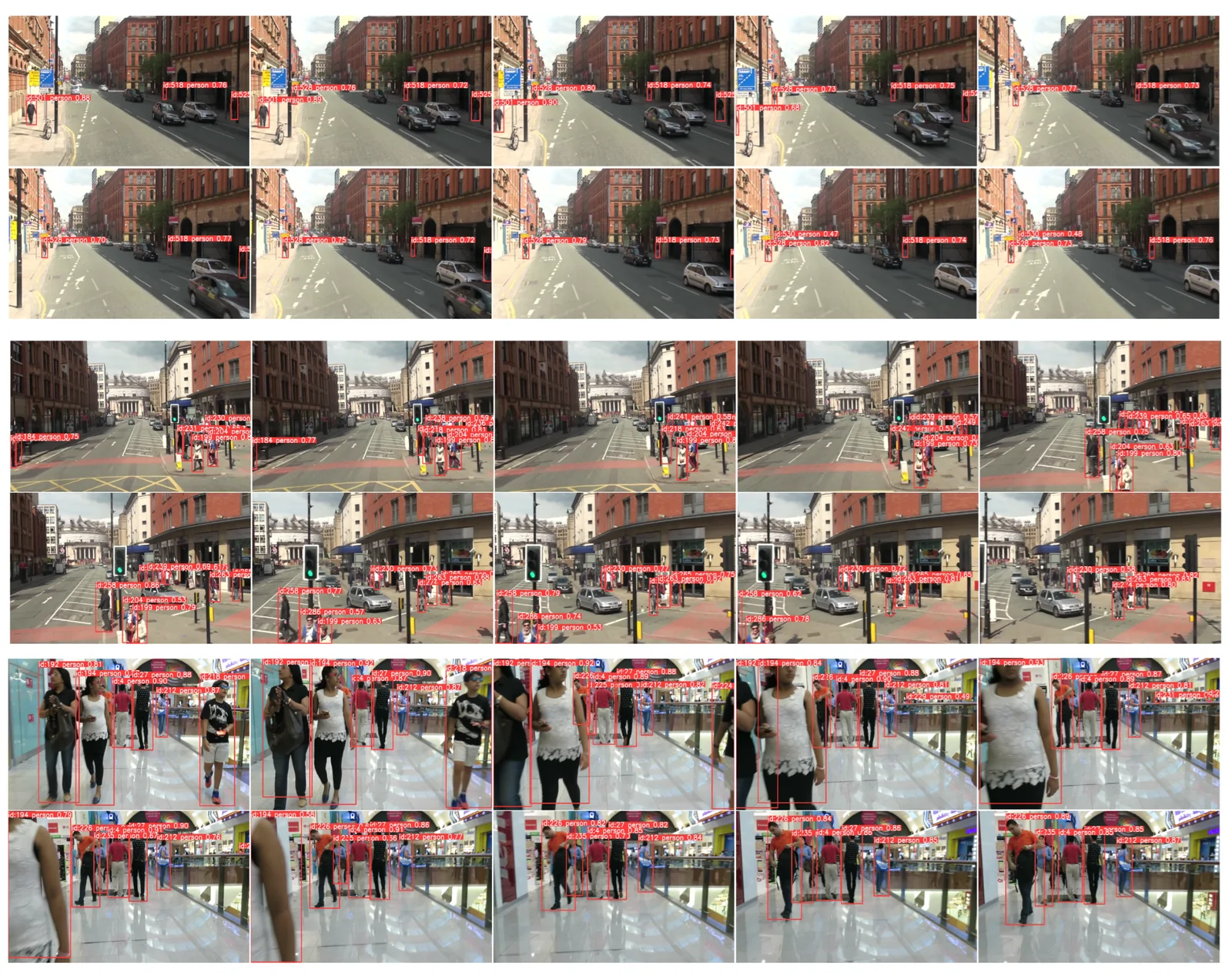
Experiment results from our proposed method on the MOT17 tracking dataset.

Figure 3 shows the visual tracking results of StrongerSORT on other test sequences of MOT17. The qualitative results demonstrate that the proposed method can effectively perform continuous multiple object tracking under various scene conditions and maintain the continuity and consistency of target identities. It also demonstrates stable association performance in complex backgrounds, scale variations, and partial occlusions, reflecting strong robustness, stability, and scene adaptability.

**Figure 4:**
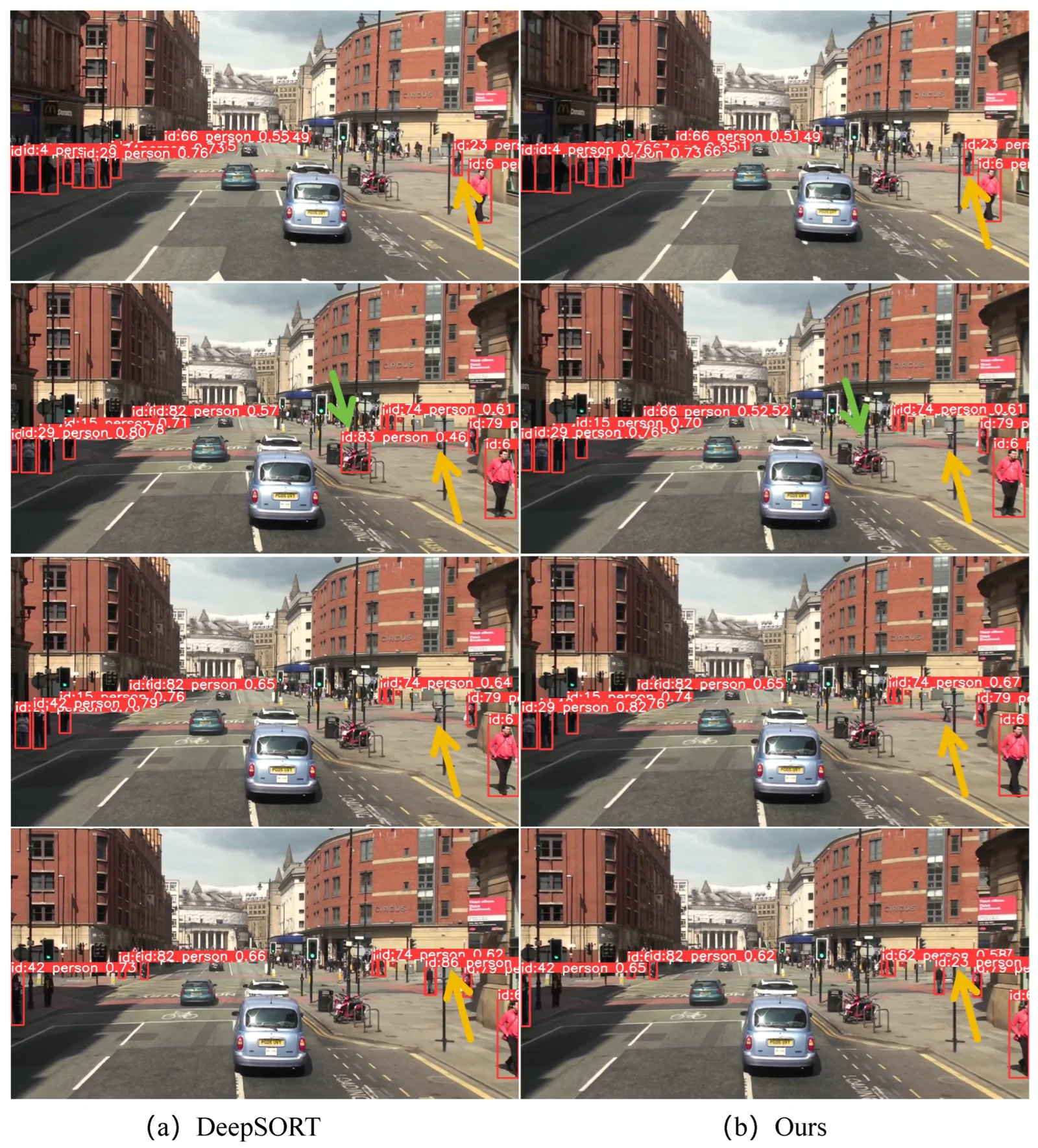
Demonstration of the qualitative comparison experiment of MOT17.

Figure 4 shows a qualitative comparison of multiple-target-tracking results between DeepSORT and the improved StrongerSORT on the same video sequence. The left column shows the tracking results of DeepSORT, and the right column shows the tracking results of StrongerSORT. As can be seen from the first line, the target indicated by the yellow arrow is identified as a pedestrian by both methods when entering the current scene, and the identification number is ID-23. As the video sequence progresses, in the second and third row, the target experienced short-term occlusion and partial disappearance due to factors such as other targets in the scene, roadside facilities, and changes in perspective. During this process, DeepSORT failed to make full use of the target’s historical appearance information and cross-frame association information. When the target reappeared in subsequent frames, its identification number in the fourth row had changed from the original ID-23 to the new ID-86, indicating a clear identity switch. In contrast, the improved StrongerSORT demonstrates a more stable ability to maintain identity in the same scenario. Although the target also went through a short period of occlusion and re-emergence, StrongerSORT was always able to correctly associate it with the original ID-23 in the corresponding results on the right, without generating a new incorrect identity assignment. This indicates that the improved tracking model is more robust in target appearance feature modeling, historical information utilization, and cross-frame data association. It can maintain a consistent judgment of the same target identity even after the target temporarily disappears, thereby effectively reducing the phenomenon of identity switching. The results in the second row on the left also show that DeepSORT misidentified a motorcycle pointed to by the green arrow in the scene as a pedestrian target and assigned it a corresponding detection box and identity information, resulting in a significant false detection. In the results for StrongerSORT on the right, no such error detection was found, indicating that StrongerSORT improves the reliability of the system in identifying real pedestrian targets while maintaining identity consistency.

### 4.3. Quantitative Results

Table 4, Table 5 and Table 6 present the comparison results of SORT [29], DeepSORT [25], ByteTrack [21], OC-SORT [23], StrongSORT [30,31], BoT-SORT [22,32], Hybrid-SORT [24], BoostTrack [32,33], and the proposed StrongerSORT on the three datasets MOT16, MOT17, and MOT20. Higher values of MOTA, IDF1, and HOTA indicate better overall tracking accuracy, identity consistency, and overall association performance, respectively. Conversely, lower values of IDs and Frag correspond to fewer identity switches and trajectory fragmentations, indicating stronger tracking continuity.

From the overall cross-dataset trends, the MOTA, IDF1, and HOTA scores of various methods on MOT17 are generally higher than those on MOT16, whereas on MOT20, overall metrics drop noticeably, and both IDs and Frag increase significantly. The observed performance difference can be primarily attributed to the varying complexity of the benchmark datasets. MOT20 contains significantly denser crowds, more severe occlusions, and more frequent interactions between targets, leading to increased detection misses and association ambiguities, which in turn cause more identity switching and trajectory breakage. In comparison, MOT16 and 17 have lower congestion levels and better target separability, resulting in higher and more stable overall tracking metrics.

In terms of overall accuracy, StrongerSORT demonstrates significant advantages in terms of identity-association quality and trajectory continuity. On MOT16, StrongerSORT achieved the highest MOTA (76.5) and HOTA (66.0), indicating more stable overall tracking quality in a balanced way, considering false positives, false negatives, and correlation errors. On MOT17, StrongerSORT’s IFD1 improved to 80.4 and HOTA reached 68.4, again outperforming the comparison methods, demonstrating its strong overall tracking capability even with more varied scene changes. On the most challenging MOT20, StrongerSORT still achieved the highest HOTA (65.7) and lowest Frag (363), but it did not achieve the highest MOTA, IDF1, or MT. This is because MOT20 contains highly crowded scenes with a high density of targets and frequent and prolonged occlusions. Meanwhile, StrongerSORT achieves IDF1 scores of 78.1 and 80.4 on MOT16 and MOT17, respectively, all of which are the highest values, indicating that it is more reliable in cross-frame identity association and can better maintain the consistency of target identity. This is one of its most prominent performance gains compared to other methods.

In terms of continuity metrics, StrongerSORT has the lowest Frag values on all three datasets, indicating that it has the fewest trajectory breaks and stronger recovery capabilities after target loss, which can significantly improve the temporal continuity of the trajectory. In terms of IDs, StrongerSORT scored 386 and 476 on MOT17 and MOT20, respectively, both of which are better than other comparison methods, indicating that its identity switching control is more effective under complex occlusion and interaction conditions. Although StrongerSORT shows slightly higher IDs than some methods on MOT16, considering that it simultaneously achieves the highest MOTA, IDF1, and HOTA scores as well as the lowest Frag, it is evident that StrongerSORT achieves a superior overall balance of “overall accuracy—identity consistency—trajectory continuity” on this dataset. That is, it significantly improves overall tracking quality without degrading trajectory continuity, while maintaining optimal identity consistency. Compared with StrongSORT, BoT-SORT, and Hybrid-SORT, StrongerSORT achieves the best performance on HOTA and Frag, while it is inferior to some competing methods on IDF1, IDs, and ML. These results indicate that StrongerSORT provides competitive overall tracking performance, although it does not achieve the best performance across all evaluation metrics. It should be noted that the current experimental setup does not use identical detection inputs. Therefore, the reported improvements reflect the combined effect of the improved YOLOv12 detector and the proposed tracking enhancements.

### 4.4. Ablation Experiments

To verify the contribution of the improved YOLOv12 modules to the model performance, we conducted ablation experiments on the SHWD dataset. The results are shown in Table 7. The baseline model, without incorporating DLKA, CIoU, and WIoU, achieves mAP0.5 and mAP0.5:0.95 of 72.6% and 42.6%, respectively. Although the inference latency is only 2.7 ms, the overall detection performance remains relatively weak. From the single-module results, introducing WIoU alone leads to the most significant improvement in recall, reaching 88.6%, indicating that this loss function is more effective in reducing missed detections. However, the precision drops to 80.9%, suggesting that using WIoU alone may introduce a certain number of false positives. In contrast, after introducing DLKA alone, the model’s P, mAP0.5, and mAP0.5:0.95 increased to 92.3%, 92.7%, and 60.2%, respectively, indicating that DLKA has the most significant effect on enhancing feature representation ability, but it also brings a certain increase in latency. The overall performance of the dual-module combinations is better than to that of single modules, indicating that structural improvements and loss function optimization exhibit strong complementarity. When DLKA is combined with CIoU or WIoU, both detection accuracy and positioning performance are further improved. Ultimately, when DLKA, CIoU, and WIoU are introduced simultaneously, the model achieves the best overall performance, with P, R, mAP0.5, and mAP0.5:0.95 reaching 92.6%, 87.9%, 93.1%, and 60.6%, respectively. In summary, DLKA is the main source of performance improvement, while CIoU and WIoU further enhance the bounding box regression and sample learning effects. The combination of the three can achieve a better balance between accuracy and real-time performance. It should be noted that the latency reported in this experiment is measured only for the detector and therefore can only confirm the low-latency performance of the detection stage. In the tracking stage, StrongerSort and StrongSort share the same order-of-magnitude time complexity with only marginal differences. The latency discrepancy of the overall tracking system mainly originates from the detector stage.

To verify the specific contributions of the NSA KF, OSNet, and EMA modules to tracking performance, we conducted further ablation experiments. As shown in Table 8, the baseline model’s MOTA, IDF1, and HOTA on the MOT17 dataset are 61.6%, 62.0%, and 53.3%, respectively. The improvement in MOTA was more significant after NSA KF was introduced separately, while IDs and Frag both decreased significantly, indicating that the improved motion prediction and state update mechanism can effectively enhance the stability of trajectory association. The improvement in IDF1 and HOTA after introducing OSNet separately is more significant, indicating that the stronger ability to express appearance features helps to improve the quality of identity differentiation and association. In contrast, the overall gain is relatively limited when EMA is introduced alone; however, it can still play a certain auxiliary role in enhancing performance. Furthermore, the results of combining any two modules are superior to those of a single module, among which the combination of NSA-KF and OSNet shows the most significant improvement, highlighting the complementary advantages of motion information and appearance information. Finally, when all three modules are integrated, the model achieves the best performance across all metrics, indicating that the proposed improvements provide strong synergistic gains.

## 5. Conclusions

This paper addresses key challenges in human tracking in complex scenarios, such as difficulty in confirming personnel identities and continuously tracking behavior. For object detection, this paper introduces deformable large-kernel attention and lightweight attention modules on the YOLOv12 baseline model to improve feature representation capabilities. At the same time, it combines GhostConv and dynamic convolution to achieve a lightweight design, and adopts an improved Weighted-CIoU loss function to optimize the bounding box regression accuracy.

The proposed StrongerSORT method, which extracts full-scale appearance features through OSNet, enhances the stability of the time dimension by combining the EMA feature moving average mechanism. Compared to traditional DeepSORT and StrongSort, StrongerSORT improves identity preservation and trajectory continuity in scenarios with complex occlusion, dense targets, and similar appearances. Experimental validation on the MOT16, MOT17, and MOT20 datasets shows that StrongerSORT provides competitive tracking performance and meets the requirements for real-time performance and deployability, providing a reliable trajectory foundation for safety supervision in complex scenarios.

The proposed method adopts a two-stage tracking-by-detection (TBD) paradigm. Similar to other TBD methods, it relies heavily on frame-by-frame detection results. Consequently, missed detections may directly interrupt target trajectories, false detections may generate spurious tracks, and inaccurate bounding-box localization may lead to association failures. Moreover, the detector and tracker cannot be jointly trained in an end-to-end manner. The detection stage does not exploit historical temporal information, while tracking performance cannot be fed back to optimize the detector. In future work, we will consider integrating detection and temporal association into a unified network for joint optimization. By exploiting global temporal features across multiple frames, the proposed framework is expected to improve identity preservation under prolonged occlusions and in densely crowded scenarios further.

## Figures and Tables

**Table 1:** NSA-KF Update Stage of Human Tracking (frame *k*).

Input	Observation $z_{k}$; Detection Confidence $c_{k}$; Predicted State ${\hat{x}}_{k|k-1}$; Predicted Covariance $P_{k|k-1}$; Observation Matrix $H_{k}$; Measurement Noise $R_{k}$
Output	Updated State ${\hat{x}}_{k|k}$; Updated Covariance $P_{k|k}$
(1) Confidence-adaptive measurement noise	${\tilde{R}}_{k}\leftarrow \left(1-c_{k}\right)R_{k}$
(2) Pre-fit residual update	${\tilde{y}}_{k}\leftarrow z_{k}-H_{k}{\hat{x}}_{k|k-1}$
(3) Residual covariance update	$S_{k}\leftarrow H_{k}P_{k|k-1}H_{k}^{⊤}+{\tilde{R}}_{k}$
(4) Kalman gain	$K_{k}\leftarrow P_{k|k-1}H_{k}^{⊤}S_{k}^{-1}$
(5) State update	${\hat{x}}_{k|k}\leftarrow {\hat{x}}_{k|k-1}+K_{k}{\tilde{y}}_{k}$
(6) Covariance update	$P_{k|k}\leftarrow \left(I-K_{k}H_{k}\right)P_{k|k-1}$

**Table 2:** Training settings.

Batch size	128
Input image size	128 × 64
Optimizer	SGD
Learning rate schedule	CosineAnnealingLR
Initial learning rate	0.01
Minimum learning rate	1 × $10^{-5}$
Epochs	100
Augmentation strategies	RandomCrop, RandomHorizontalFlip, ColorJitter, Normalize, RandomErasing
ReID training dataset	Market1501
Matching threshold	0.2
IoU matching threshold	0.7
EMA momentum	0.9
SGD momentum	0.9
Weight decay	0.0005
Label smoothing	0.1
Dropout	0.5
Maximum lost frames	60
Maximum unmatched predictions	7
Track initialization frames	2
Feature memory bank size	100

**Table 3:** Network architecture configuration of the backbone.

Name	Patch Size/Stride	Output Size (C×H×W)
Input	–	3×640×640
Conv (P1/2)	3×3/2	64×320×320
Conv (P2/4)	3×3/2	128×160×160
C3k2_DCNv3_DLKA × 2	–	256×160×160
Conv (P3/8)	3×3/2	256×80×80
C3k2_DCNv3_DLKA × 2	–	512×80×80
Conv (P4/16)	3×3/2	512×40×40
C3k2_DCNv3_DLKA × 4	–	512×40×40
Conv (P5/32)	3×3/2	1024×20×20
C3k2_DCNv3_DLKA × 4	–	1024×20×20

**Table 4:** Comparison of Different Algorithms on MOT16 tracking datasets.

Model	MOTA (↑)	IDF1 (↑)	HOTA (↑)	IDs (↓)	Frag (↓)	MT (↑)	ML (↓)
SORT [29]	53.1	45.8	40.6	1128	1283	18.7	34.6
DeepSORT [25]	60.2	58.2	51.8	892	839	25.9	27.8
ByteTrack [21]	71.5	75.9	64.8	442	356	41.6	17.2
OC-SORT [23]	70.3	72.0	62.5	**427**	407	38.9	18.5
StrongerSORT	**76.5**	**78.1**	**66.0**	441	**306**	**44.2**	**15.9**

**Table 5:** Horizontal Comparison of Different Algorithms on MOT17.

Model	MOTA (↑)	IDF1 (↑)	HOTA (↑)	IDs (↓)	Frag (↓)	MT (↑)	ML (↓)
SORT [29]	58.3	55.2	45.7	1294	1451	22.6	31.8
DeepSORT [25]	61.6	62.0	53.3	852	946	28.9	25.4
ByteTrack [21]	76.8	77.6	66.1	**364**	284	43.7	14.2
OC-SORT [23]	64.4	74.7	65.2	397	311	39.5	16.8
StrongSORT [30,31]	78.3	78.5	63.5	1446	1866	53.6	13.9
BoT-SORT [22,32]	**80.6**	79.5	64.6	1257	1803	**54.4**	16.2
Hybrid-SORT [24]	76.4	76.2	62.5	-	-	-	-
BoostTrack [32,33]	80.5	80.2	65.4	1104	1791	50.1	16.7
StrongerSORT	79.0	**80.4**	**68.4**	386	**253**	45.8	**13.1**

**Table 6:** Comparison of Different Algorithms on the MOT20 tracking datasets.

Model	MOTA (↑)	IDF1 (↑)	HOTA (↑)	IDs (↓)	Frag (↓)	MT (↑)	ML (↓)
SORT [29]	45.2	40.7	35.6	1982	1952	15.4	42.8
DeepSORT [25]	50.7	50.5	45.2	1406	1479	22.7	34.9
ByteTrack [21]	73.9	72.7	63.1	517	476	39.8	18.7
OC-SORT [23]	69.7	70.5	62.2	588	501	37.6	20.4
StrongSORT [30,31]	72.2	75.9	61.5	1066	1003	**62.1**	14.9
BoT-SORT [22,32]	**77.7**	**76.3**	62.6	1212	-	-	-
Hybrid-SORT [24,34]	76.4	76.2	62.5	1306	3517	54.8	10.1
BoostTrack [33,34]	76.4	76.5	63	992	966	57.9	**9.5**
StrongerSORT	72.5	74.4	**65.7**	**476**	**363**	41.3	16.5

**Table 7:** Ablation study of improved model on SHWD dataset.

DLKA	CIoU	WIoU	P (%)	R (%)	mAP_0.5_ (%)	mAP_0.5:0.95_ (%)	Latency (ms)
-	-	-	89.2	65.6	72.6	42.6	**2.7**
-	-	✓	80.9	**88.6**	85.0	43.4	3.5
-	✓	✓	91.5	86.4	92.3	59.6	3.0
✓	-	-	92.3	86.8	92.7	60.2	4.9
✓	✓	-	92.4	87.1	92.9	60.4	4.7
✓	-	✓	92.0	87.3	92.8	60.3	4.8
✓	✓	✓	**92.6**	87.9	**93.1**	**60.6**	4.6

**Table 8:** Ablation results on the MOT17 tracking dataset.

NSA KF	OSNet	EMA	MOTA (↑)	IDF1 (↑)	HOTA (↑)	IDs (↓)	Frag (↓)
-	-	-	61.6	62.0	53.3	852	946
✓	-	-	65.1	65.8	56.1	738	812
-	✓	-	64.5	67.2	56.5	772	845
-	-	✓	63.2	64.1	54.6	818	901
✓	✓	-	74.2	76.3	64.5	498	432
✓	-	✓	70.8	72.4	60.9	612	571
-	✓	✓	71.9	74.0	61.8	576	523
✓	✓	✓	**79.0**	**80.4**	**68.4**	**386**	**253**

## Data Availability

The datasets in the paper are from public datasets which can be downloaded from https://opendatalab.com/MOT20 (accessed on 10 September 2026).

## Abbreviations

The following abbreviations are used in this manuscript:

PMS	Personnel Monitoring System
YOLO	You Only Look Once
NSA	Noise-Scale Adaptive
OSNet	Omni-Scale Network
HOG	Histogram of Oriented Gradient
LBP	Local Binary Pattern
SVM	Support Vector Machine
VGG	Visual Geometry Group
COCO	Common Objects in Context
GPU	Graphic Processing Unit
MOT	Multiple-Object Tracking
DBT	Tracking By Detection
SORT	Simple Online and Realtime Tracking
NMS	Non-Maximum Suppression
DLKA	Deformable Large-Kernel Attention
MOTA	Multiple-Object Tracking Accuracy
HOTA	Higher-Order Tracking Accuracy
SHWD	Safety Helmet-Wearing Dataset
